# *Staphylococcus aureus* screening and preoperative decolonisation with Mupirocin and Chlorhexidine to reduce the risk of surgical site infections in orthopaedic surgery: a pre-post study

**DOI:** 10.1186/s13756-024-01432-2

**Published:** 2024-07-11

**Authors:** Antoine Portais, Meghann Gallouche, Patricia Pavese, Yvan Caspar, Jean-Luc Bosson, Pascal Astagneau, Regis Pailhé, Jérôme Tonetti, Brice Rubens Duval, Caroline Landelle

**Affiliations:** 1 Infectious and Tropical Diseases Unit, Grenoble Alpes University Hospital, Grenoble, France; 2 Hospital Hygiene Unit, Grenoble Alpes University Hospital, Grenoble, France; 3 Univ. Grenoble Alpes, CNRS, Grenoble INP, TIMC, Grenoble, France; 4 Bacteriology Laboratory, Grenoble Alpes University Hospital, Grenoble, France; 5 Univ. Grenoble Alpes, CEA, CNRS, IBS, Grenoble, France; 6 Public Health Department, Grenoble Alpes University Hospital, Grenoble, France; 7 Centre for the prevention of healthcare associated infections (CPIAS), Paris, France; 8 Pierre Louis Institute of Epidemiology and Public Health, Sorbonne University, INSERM, Paris, France; 9 Orthopaedic Surgery Unit, Clinique Aguiléra, Ramsay Santé, Biarritz, France; 10 Department of Orthopaedic Surgery, Grenoble Alpes University Hospital, Grenoble, France; 11 Department of Osteoarthritis and Sport Surgery, Grenoble Alpes University Hospital, Grenoble, France

**Keywords:** *Staphylococcus aureus*, Screening, Decolonisation, Surgical site infection, Mupirocin, Chlorhexidine

## Abstract

**Background:**

Nasal carriage of *Staphylococcus aureus* is a risk factor for surgical site infections (SSI) in orthopaedic surgery. The efficacy of decolonisation for *S. aureus* on reducing the risk of SSI is uncertain in this speciality. The objective was to evaluate the impact of a nasal screening strategy of *S. aureus* and targeted decolonisation on the risk of *S. aureus* SSI.

**Methods:**

A retrospective pre-post and here-elsewhere study was conducted between January 2014 and June 2020 in 2 adult orthopaedic surgical sites (North and South) of a French university hospital. Decolonisation with Mupirocin and Chlorhexidine was conducted in *S. aureus* carriers starting February 2017 in the South site (intervention group). Scheduled surgical procedures for hip, knee arthroplasties, and osteosyntheses were included and monitored for one year. The rates of *S. aureus* SSI in the intervention group were compared to a historical control group (South site) and a North control group. The risk factors for *S. aureus* SSI were analysed by logistic regression.

**Results:**

A total of 5,348 surgical procedures was included, 100 SSI of which 30 monomicrobial *S. aureus* SSI were identified. The preoperative screening result was available for 60% (1,382/2,305) of the intervention group patients. Among these screenings, 25.3% (349/1,382) were positive for *S. aureus* and the efficacy of the decolonisation was 91.6% (98/107). The rate of *S. aureus* SSI in the intervention group (0.3%, 7/2,305) was not significantly different from the historical control group (0.5%, 9/1926) but differed significantly from the North control group (1.3%, 14/1,117). After adjustment, the risk factors of *S. aureus* SSI occurrence were the body mass index (ORa_per unit_, 1.05; 95%CI, 1.0-1.1), the Charlson comorbidity index (ORa_per point_, 1.34; 95%CI, 1.0–1.8) and operative time (ORa_per minute_, 1.01; 95%CI, 1.00–1.02). Having benefited from *S. aureus* screening/decolonisation was a protective factor (ORa, 0.24; 95%CI, 0.08–0.73).

**Conclusions:**

Despite the low number of SSI, nasal screening and targeted decolonisation of *S. aureus* were associated with a reduction in *S. aureus* SSI.

## Background

Surgical site infections (SSI) are a major complication in orthopaedic surgery. In a systematic review, the median incidence of SSI in orthopaedic surgery was estimated to be 2.7%. *Staphylococcus aureus* concerned 59% of them [1]. In France, according to the national program for investigation and surveillance of healthcare-associated infection (RAISIN) from 2018, the median incidence was estimated to be 1.4% of which 37.4% were linked to *S. aureus* [2].

Nasal carriage of *S. aureus* is a risk factor of SSI in orthopaedic surgery [3, 4]. But the efficacy of *S. aureus* decolonisation on reducing the risk of SSI is uncertain in this speciality. Indeed, a meta-analysis conducted by the World Health Organisation (WHO) in 2016, including 6 randomised controlled studies in several surgical specialities and evaluating the efficacy of Mupirocin (+/-Chlorhexidine), showed a significant reduction in the risk of *S. aureus* SSI (Odds Ratio[OR]: 0.46; 95% Confidence interval [95%CI]: 0.31–0.69) [5]. However, only 2 studies had included orthopaedic surgery patients. Since 2016, two other randomised controlled studies were conducted with orthopaedic surgery patients, and evaluated the efficacy of decolonisation of *S. aureus* with Mupirocin and Chlorhexidine on the occurrence of *S. aureus* SSI. These studies did not find any significant difference between the intervention groups and the groups without decolonisation, respectively 3.4% (3/89) vs. 4.3% (6/139) [6] and 0.4% (1/232) vs. 0.4% (1/233) [7].

Despite the low number of studies with a high level of proof, the WHO recommends *S. aureus* nasal screening and decolonisation in orthopaedic surgery since 2016 [5]. In France, in the latest recommendations from 2013, it is not recommended to decolonise patients in orthopaedic surgery, due to insufficient data [8].

Between January 2012 and April 2015, a study [9] was conducted at the Grenoble Alps University Hospital (CHUGA), which found a rate of SSI in orthopaedic surgery of 1.8%; 0.7% were monomicrobial *S. aureus* SSI. The risk factors for *S. aureus* SSI identified were smoking, a National Nosocomial Infections Surveillance (NNIS) score ≥1 and the absence of a preoperative shower. The nasal carriage of *S. aureus* was not evaluated. *S. aureus* screening and targeted decolonisation by nasal applications of Mupirocin ointment and showers with Chlorhexidine was implemented in February 2017, for scheduled orthopaedic surgical procedures, in one of the 2 orthopaedic surgery sites (South site) of the CHUGA.

The main objective of this study was to evaluate the impact of implementing the strategy for screening and targeted decolonisation on the risk of monomicrobial *S. aureus* SSI after scheduled orthopaedic surgery. The secondary objectives were to evaluate the impact of the strategy on all SSI regardless of the microorganism, on SSI linked to cutaneous commensal flora microorganisms (CCFM) and to evaluate the individual risk factors associated with the occurrence of *S. aureus* SSI.

## Methods

### Location

CHUGA is a French university hospital with 2,133 beds and places (last available data in 2018) distributed across various sites. Within CHUGA, there are two orthopaedic surgery departments treating patients on 2 geographically different sites (South and North) located around 12 km apart. There are 57 beds and 7 surgeons for the South site and 62 beds and 6 surgeons for the North site; surgeons belonging solely to one site without overlapping between the two sites. Between 2014 and 2020, on average, 3,844 surgical procedures were performed each year on the South site and 1,293 on the North site.

### Design of the study and endpoints

This was a retrospective, real-life, monocentric pre-post and here-elsewhere study. All the surgical procedures performed in the South and North sites between 01.01.2014 and 30.06.2020 were selected. The North control group was defined in the North hospital, including surgical procedures performed between 01.01.2014 and 30.06.2020. The historical control group was defined in the South Hospital, including the surgical procedures performed before the implementation of the strategy for screening and decolonisation of *S. aureus* between 01.01.2014 and 31.01.2017. The intervention group was defined in the South Hospital, including the surgical procedures performed after the implementation of the strategy between 01.02.2017 and 30.06.2020*.*

The selection criteria were the scheduled surgical procedures (days between admission and the procedure ≥10), carried out on subjects over 16 years old, and defined by the RAISIN protocol [10]. These surgical procedures were knee and hip arthroplasties, revision of knee and hip arthroplasties, osteosyntheses of the upper end of the femur and other osteosyntheses (except for cranial and vertebral). Surgical procedures on the hand, external fixator placements and surgical procedures for an infection were excluded.

The primary endpoint was the rate of monomicrobial *S. aureus* SSI. The secondary endpoints were the global rate of SSI regardless of microorganisms and the rate of CCFM SSI only. The CCFM included *Cutibacterium spp*, *Corynebacterium spp*, *S. aureus* and coagulase-negative *Staphylococci* (CoNS).

### Strategy for screening and decolonisation

The strategy was implemented in the South Hospital, starting in February 2017. Between February 2017 and December 2019, during consultation with the surgeon where the surgery was scheduled, a prescription was given to the patient for the screening for methicillin-sensitive (MSSA) and methicillin-resistant (MRSA) *S. aureus* either in a medical laboratory, or at the CHUGA collection centre. Since January 2019, this screening was done at CHUGA by a nurse if the surgery was scheduled within the following 3 months, or a prescription was given to the patient for surgical procedures scheduled more than 3 months after consultation. It was recommended that screening be carried out no more than 3 months before surgery. At CHUGA, the collection was taken via nasal swabbing with an E-swab then was cultured on Columbia blood agar. The identification was done by MALDI-TOF mass spectrometry.

If the screening was positive, a prescription for decolonisation was given to the patient. It had to be done at best during the 5 days preceding the surgery, or started at the latest the day before the surgery. The protocol included a calcium Mupirocin 2% ointment: 1 application in the 2 nostrils with a nostril massage, twice a day for 5 days, and Chlorhexidine digluconate 4%: 1 shower/day for 5 days and 2 to 3 shampoos distributed over 5 days. Patients were also to use a clean bath towel before the first shower, and change the bed sheets on the 1^st^ day of treatment. When the strategy was first implemented, a nasal swab for *S. aureus* detection was carried out in the perioperative period, between D-1 and D+7 of the surgery to evaluate the efficacy of the decolonisation. This control measure was later dropped, so the efficacy was evaluated only on a portion of the study population.

### SSI prevention measures

Conventional SSI prevention measures were recommended identically in both sites and in both study periods. They included 2 preoperative antiseptic showers, carried out the day before the surgery and the day of surgery. Hair removal using depilatory cream or clippers was left to the discretion of the surgeon. Antibiotic prophylaxis was carried out in the operating theatre, according to the French national recommendations [11]. They recommended cefazolin for most of the surgical procedures, or vancomycin in case of allergy or MRSA colonisation. Note that the administration time changed in 2018, from 1 hour to 30 minutes before the surgery.

### SSI definition and data collection

We used the SSI definition of the Centres for Disease Control and Prevention (CDC) [12], but with a postoperative period set to 1 year after the surgery. SSI were identified either through a semi-automated surveillance program using surgery data, microbiological data, antibiotic prescriptions and hospitalisation data [9], or they were reported by colleagues for patients who were subsequently treated in another facility.

For all surgical procedures, the following data was collected: age and gender of the patient, Charlson comorbidity index (CCI) [13], body mass index (BMI), date, type and site of the surgery (North or South), time between admission and the surgery, Altemeier classification (stratification of the postoperative SSI risk depending on the type of surgery) [14], American Society of Anesthesiologists score (ASA), and length of the surgical procedure.

For surgical procedures followed by an SSI, the following additional data were collected: site and type of SSI (superficial or deep), microorganisms responsible, active smoking (or quitting <1 month), alcoholism, intravenous drug abuse, high blood pressure, immunosuppression, negligence, carrying out of an antiseptic shower on D0 of the surgery, position in the daily surgical schedule, number of people in the theatre, presence of postoperative haematoma, postoperative anticoagulation, adequacy of the preoperative antibiotic prophylaxis (suitable molecule) and compliance with postoperative recommendations. For the orthopaedic procedures of the South site, the date of the nasal screening samples and the result were collected. If the result was positive, details were provided on the MSSA or MRSA resistance phenotype.

### Statistics

The rates of monomicrobial *S. aureus* SSI, all SSI regardless of microorganisms, and CCFM were expressed as cumulative incidence rates per period or per year (number of SSI for 100 surgical procedures, percentage).

Quantitative variables were expressed as medians and interquartiles (Q1-Q3), and qualitative variables were expressed as numbers (n) and percentages (%). In bivariate analysis, the groups were compared by means of the Mann–Whitney test, Pearson Chi-squared test or Fisher’s exact test. Evolution of the *S. aureus* SSI incidence rate over the years was analysed by testing the slope of the linear regression.

Multivariable logistic regression analysis of the primary endpoint with adjustment on the risk factors of *S. aureus* SSI was also conducted. The control group included surgery procedures without SSI. The following risk factors for monomicrobial *S. aureus* SSI were evaluated in bivariate analysis: age, gender, BMI, CCI, ASA score, length of surgery, Altemeier classification, length of hospitalisation before surgery, type of surgery, site of surgery (North or South), presence of preoperative screening for *S. aureus*. Variables with a p-value <0.05 (to limit the number of variables included considering the low number of events) were considered for inclusion in the multivariate analysis, the presence of preoperative screening was forced in the model. A stepwise approach was used to select the regression model and a p-value >0.05 was defined to remove variables from the final model. The statistical analyses were carried out with STATA version 17.0 (StataCorp. 2021. Stata Statistical Software: Release 17. College Station, TX:StataCorp LLC).

### Ethics

The database was declared to the CHUGA Data Protection Officer. The study was authorised by the local clinical research department on 04.02.2022. Data are reported in accordance with STROBE statement for observational studies [15].

## Results

### General points

**Fig. 1:**
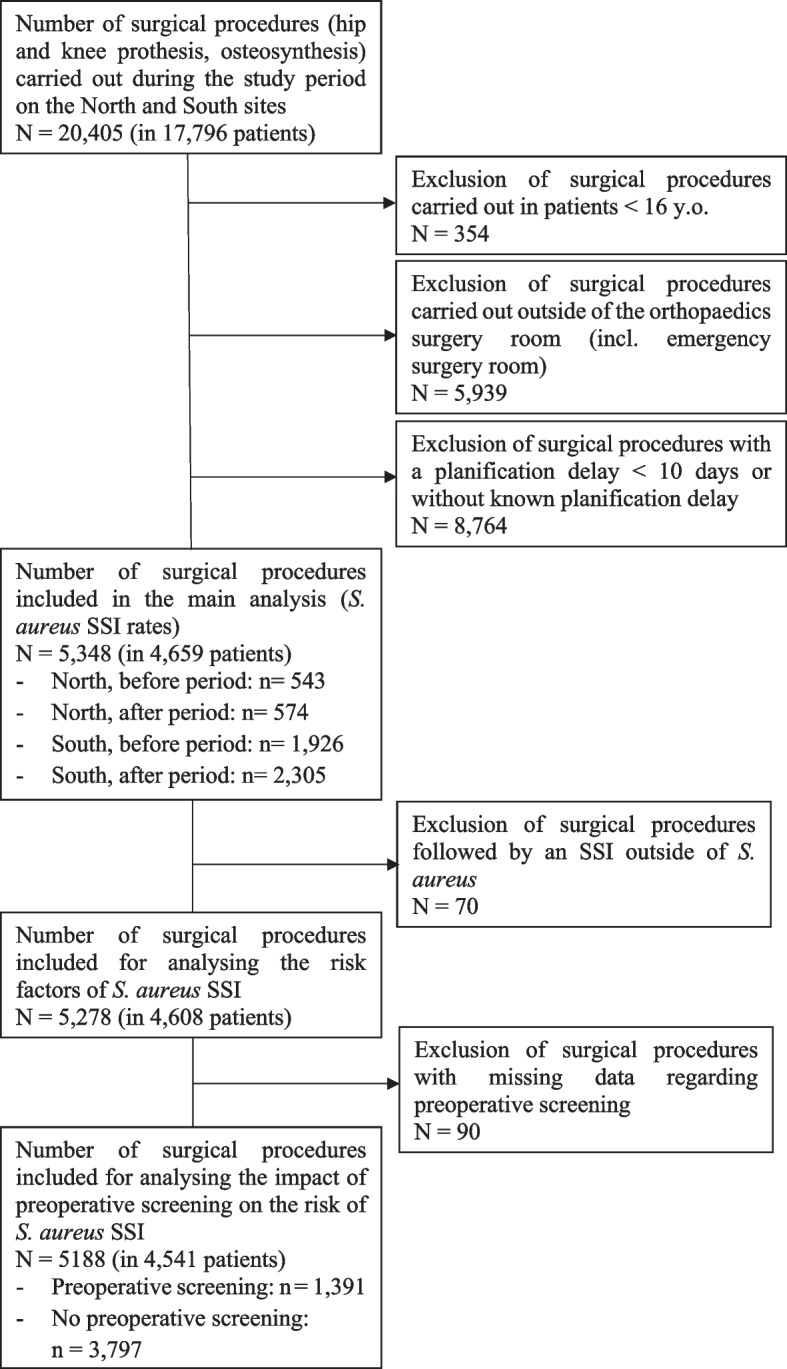
Flow chart

Among the 20,051 surgical procedures that were performed in 17,445 patients over 16 years old, a total of 5,348 scheduled surgical procedures in 4,659 patients were included in the analysis: 1,117 on the North site (543 in the period before and 574 in the period after) and 4,231 on the South site (1,926 in the period before and 2,305 in the period after) (Fig. 1).

### Description of the population

#### General description

For all the surgical procedures, the median age of the patients was 68 years, 44.2% (2,364/5,348) were male, and the majority of procedures were knee prostheses (46.0%, 2,458/5,348) (Table 1).

**Table 1:** Description of the characteristics of the surgical procedures according to the site and the period

**Variables**		**Total**	**North Site**				**South Site**			
		***n*= 5348**	**North Total** **n= 1117**	**01.01.2014 – 31.01.2017** ***n*= 543**	**01.02.2017 – 30.06.2020** ***n*= 574**	***P* value**	**South Total** ***n*= 4231**	**01.01.2014 – 31.01.2017** ***n*= 1926**	**01.02.2017 – 30.06.2020** ***n*= 2305**	***P* value**
**Age in years, median (Q1-Q3)**		68 (60 – 76)	64 (51 – 74)	64 (51 – 74)	64 (50 – 75)	0.561	68 (61 – 76)	69 (61 – 76)	69 (61 – 76)	0.441
**Male gender, n(%)**		2364 (44.2)	573 (51.3)	276 (50.8)	297 (51.7)	0.760	1791 (42.3)	830 (43.1)	961 (41.7)	0.358
**CCI ,median (Q1-Q3)**		0 (0 – 1)	0 (0 – 1)	0 (0 – 1)	0 (0 – 1)	0.595	0 (0 – 1)	0 (0 – 1)	0 (0 – 1)	**0.014**
**BMI in kg/m2** **median (Q1-Q3)**		27.7 (23.3 – 32.47)	27.2 (22.9 – 32.0)	29.2 (24.9 – 32.6)	27 (22.8 – 31.9)	0.162	27.7 (23.4 – 32.7)	28.0 (23.4 – 32.5)	27.7 (23.4 – 32.7)	0.832
**Altemeier classification, n(%)**	**1**	5308 (99.3)	1081 (96.8)	524 (96.5)	557 (97.0)	0.611	4227 (99.9)	1924 (99.9)	2303 (99.9)	0.857
	**2**	40 (0.7)	36 (3.2)	19 (3.5)	17 (3.0)		4 (0.1)	2 (0.1)	2 (0.1)	
**ASA score, n (%)**		*n*=4640	*n*= 909	*n*= 371	*n*= 539	0.398	*n*=3730	*n*= 1622	*n*= 2108	**< 0.001**
	**1**	896 (19.3)	197 (21.7)	73 (20.6)	124 (22.3)		699 (18.7)	340 (21.8)	359 (16.5)	
	**2**	2699 (58.2)	451 (49.6)	188 (53.1)	264 (47.4)		2248 (60.3)	921 (59.0)	1327 (61.2)	
	**3**	1020 (22.0)	246 (27.1)	88 (24.9)	158 (28.5)		774 (20.8)	295 (18.9)	479 (22.1)	
	**4**	24 (0.5)	15 (1.6)	5 (1.4)	10 (1.8)		9 (0.2)	4 (0.3)	5 (0.2)	
**Time between admission and the surgery in hours, median (Q1-Q3)**		17 (16 – 19)	19 (17-22)	19 (17 - 21)	20 (18 – 23)	**< 0.001**	17 (16 – 19)	17 (16 – 19)	17 (16 – 19)	0.218
**Duration of surgery in minutes, median (Q1-Q3)**		68 (52 – 89)	82 (62 – 114)	77.5 (57 - 109)	87 (66 – 120)	**< 0.001**	66 (50 – 83)	64 (47 – 83)	67 (53 - 82)	**< 0.001**
**Type of surgery,** **n (%)**	**OS of the UEF**	20 (0.4)	11 (0.98)	3 (0.6)	8 (1.4)	0.089	9 (0.2)	7 (0.4)	2 (0.1)	0.090
	**Other OS**	393 (7.3)	292 (26.1)	149 (27.4)	143 (24.9)		101 (2.4)	40 (2.1)	61 (2.7)	
	**Knee prostheses**	2458 (46.0)	234 (20.9)	127 (23.4)	107 (18.6)		2224 (52.6)	998 (51.8)	1226 (53.2)	
	**Hip prostheses**	1938 (36.2)	392 (35.1)	185 (34.1)	207 (36.1)		1546 (36.5)	733 (36.1)	813 (35.3)	
	**Revision of knee prosthesis**	190 (3.6)	37 (3.3)	17 (3.1)	20 (3.5)		153 (3.6)	63 (3.3)	90 (3.9)	
	**Revision of hip prosthesis**	349 (6.5)	151 (13.5)	62 (11.4)	89 (15.5)		198 (4.7)	85 (4.4)	113 (4.9)	
***S. aureus* SSI, n (%)**		30 (0.6)	14 (1.3)	10 (1.8)	4 (0.7)	0.086	16 (0.4)	9 (0.5)	7 (0.3)	0.388
**CCFM SSI, n (%)**		43 (0.8)	21 (1.9)	13 (2.4)	8 (1.4)	0.219	22 (0.5)	12 (0.6)	10 (0.4)	0.394
**All SSI, n (%)**		100 (1.9)	54 (4.8)	24 (4.4)	30 (5.2)	0.619	46 (1.1)	18 (0.9)	28 (1.2)	0.381

Legend: The *p*-values correspond to the comparisons of the periods before (01/01/2014–31/01/2017) and after (01/02/2017–30/06/2020), within the North site then the South site

*ASA* American Society of Anesthesiologists, *BMI* Body mass index, *CCFM* Cutaneous commensal flora microorganisms, *OS* Osteosynthesis, *SSI* Surgical site infection, *UEF* Upper end of the femur

Within the North site, the two pre-post periods were comparable except for the operative time (*p*<0.001) and the time between admission and surgery (*p*<0.001) which were longer for the period afterwards (Table 1). Within the South site, the two periods were also comparable, except for the operative time which was longer (*p*<0.001), the ASA score (*p*<0.001) and the CCI (*p*=0.014) which were higher for the period afterwards (Table 1).

In comparison with the intervention group, the patients of the North control group were significantly younger (*p*<0.001), more often male (*p*<0.001), had a higher Altemeier classification (*p*<0.001), higher ASA scores (*p*<0.001), a longer time between admission and surgery (*p*<0.001), longer operative times (*p*<0.001), more procedures for osteosyntheses and fewer procedures for knee prostheses (*p*<0.001).

#### SSI description

**Fig. 2:**
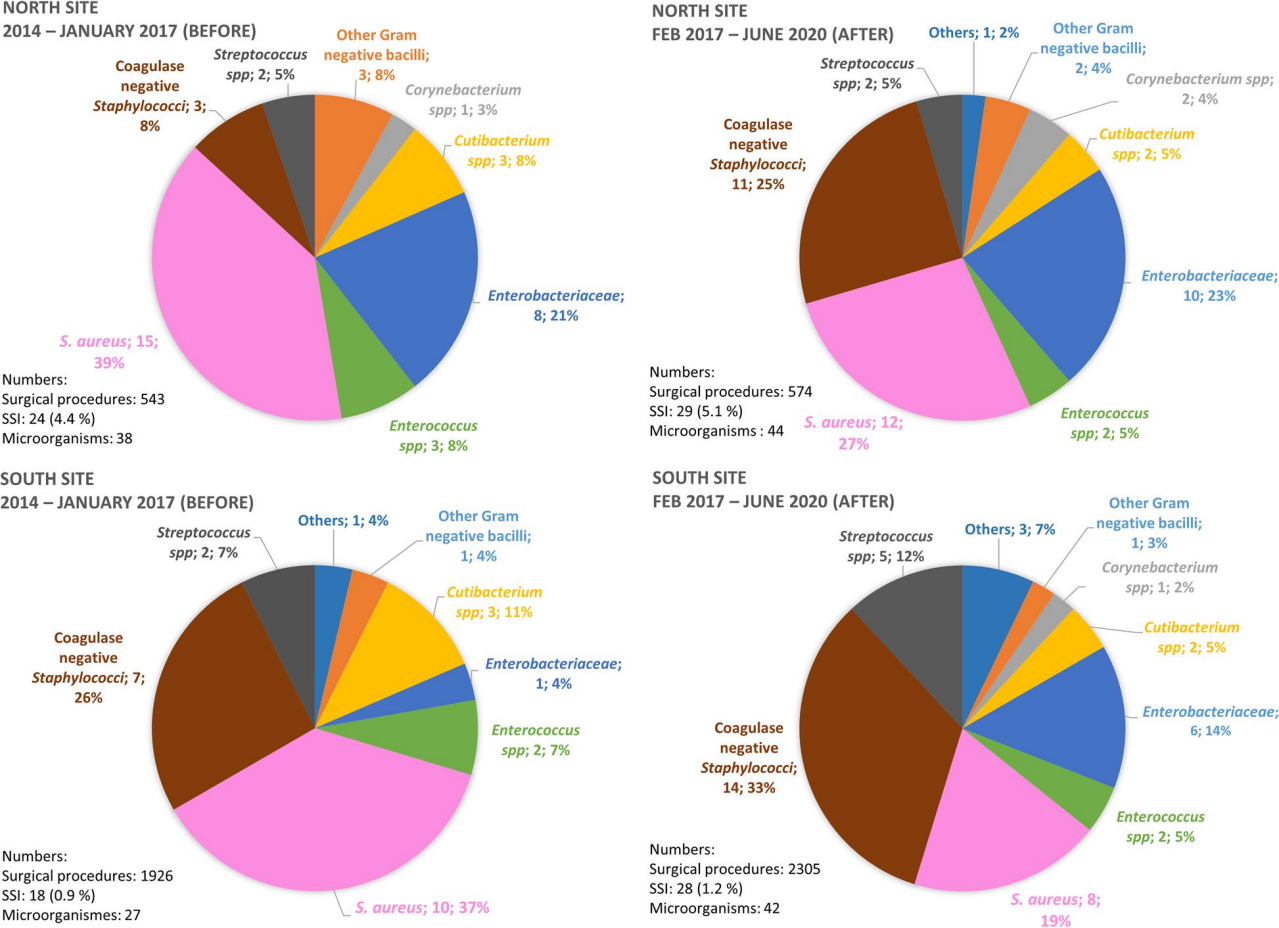
Distribution of the microorganisms involved in SSI, according to the site and the period. Legend: SSI: Surgical site infection. An SSI of the North Site of the period after without a microorganism identified was not included in this figure

For the entire population of the study, 1.9% (100/5348) surgical procedures were followed by SSI (Table 1). Among all the SSI, the median time before the occurrence was 29.5 days and 90 (90.0%) were deep SSI. Thirty (30.0%) SSI were monomicrobial *S. aureus* SSI, of which 86.7% (26/30) were deep. For one SSI, no microorganism was identified. The characteristics of patients with SSI are summarised in Table 2. *S. aureus* was the predominant microorganism, except for the intervention group (19%, 8/42) as shown in Fig. 2. For both South and North sites, there seems to be a decrease in the proportion of *S. aureus* in the period after and an increase in the proportion of CoNS.

**Table 2:** Description of the surgical site infections according to the site and the period

**Variables**		**SSI**								
		**Total**	**North**				**South**			
		***n* = 100**	**North Total** ***n*= 54**	**2014 – 2017** ***n*= 24**	**2017 – 2020** ***n*= 30**	***P* value**	**South Total** ***n*= 46**	**2014 – 2017** ***n*= 18**	**2017 – 2020** ***n*= 28**	***P* value**
**Age (in years), median (Q1-Q3)**		66 (56.5 – 74)	65 (49.5 – 72.5)	59.5 (45.5 – 70.5)	66 (51 – 75)	0.240	68.5 (60.25 – 74)	72 (64 – 78)	66 (59 (71.5)	0.057
**Male gender, n(%)**		61 (60.0)	33 (60.0)	12 (50.0)	21 (70.0)	0.134	28 (60.9)	11 (61.1)	17 (60.7)	0.979
**BMI (in kg/m2), median (Q1-Q3)**		28.9 (25.3 – 33.6)	28.8 (24.9 – 32.9)	30.2 (25.4 – 35.4)	28.1 (24.8 – 32.1)	0.134	29.6 (26.4 – 34.8)	28.4 (26.4 – 31.1)	31.3 (26.7 – 35.7)	0.405
**CCI, median (Q1-Q3)**		1 (0 – 2)	0 (0 – 2)	0.5 (0 – 2.5)	0 (0 – 2)	0.300	1 (0 – 2)	1 (0 – 2)	1 (0 – 2.5)	0.615
**ASA score, n (%)**	**1**	18 (18.0)	11 (20.4)	6 (25.0)	5 (16.7)	0.668	7 (15.2)	1 (5.6)	6 (21.4)	0.078
	**2**	41 (41.0)	21 (38.9)	8 (33.3)	13 (43.3)		20 (43.5)	6 (33.3)	14 (50.0)	
	**3**	40 (40.0)	22 (40.7)	10 (41.7)	12 (40.0)		18 (39.1)	11 (61.1)	7 (25.0)	
	**4**	1 (1.0)	0	0	0		1 (2.2)	0	1 (3.6)	
**Altemeier classification, n (%)**	**1**	99 (99.0)	53 (98.2)	23 (95.83)	30 (100)	0.444	46 (100)	18 (100)	28 (100)	-
	**2**	1 (1.0)	1 (1.8)	1 (4.2)	0		0	0	0	
**Time between admission and the surgery (in hours), median (Q1-Q3)**		18 (17 – 20)	19 (17 – 21)	19 (17 – 21.5)	19 (17 – 22)	0.680	17 (17 – 19)	17 (17 – 19)	17 (17 – 18.5)	0.881
**Duration of the surgery (in minutes), median (Q1-Q3)**		85 (65 – 125)	113 (75 – 152.75)	127 (88 – 153.5)	87 (68 – 144)	0.183	72 (59.3 – 100.3)	75 (65 – 106)	71 (58 – 81)	0.350
**Type of surgery n (%)**	**OS of the UEF**	0	0	0	0	**0.038**	0	0	0	0.074
	**Other OS**	17 (17.0)	14 (25.9)	9 (37.5)	5 (16.7)		3 (6.5)	2 (11.1)	1 (3.6)	
	**Knee prostheses**	37 (37.0)	12 (22.2)	8 (33.3)	4 (13.3)		25 (54.3)	11 (61.1)	14 (50.0)	
	**Hip prostheses**	28 (28.0)	15 (27.8)	5 (20.8)	10 (33.3)		13 (28.3)	2 (11.1)	11 (39.3)	
	**Revision of knee prosthesis**	4 (4.0)	2 (3.7)	0	2 (6.7)		2 (4.4)	2 (11.1)	0	
	**Revision of hip prosthesis**	14 (14.0)	11 (20.4)	2 (8.3)	9 (30.0)		3 (6.5)	1 (5.6)	2 (7.1)	
**Smoking, n (%)**		*n*= 98				0.079	*n*= 44		*n*= 26	0.682
		19 (19.4)	12 (22.2)	8 (33.3)	4 (13.3)		7 (15.9)	2 (11.1)	5 (19.2)	
**Alcoholism, n (%)**		*n* = 99				1.000	*n*= 45		*n*= 27	1.000
		4 (4.0)	1 (1.9)	0	1 (3.3)		3 (6.7)	1 (5.6)	2 (7.4)	
**IV drug abuse, n (%)**		0								
**Presence of HBP, n (%)**		58 (58.0)	26 (48.2)	10 (41.7)	16 (53.3)	0.394	32 (69.6)	14 (77.8)	18 (64.3)	0.332
**Presence of diabetes, n (%)**		26 (26.0)	12 (22.2)	8 (33.3)	4 (13.3)	0.079	14 (30.4)	8 (44.4)	6 (21.4)	0.098
**Presence of immunosuppression, n (%)**		4 (4.0)	1 (1.8)	0	1 (3.3)	1.000	3 (6.5)	2 (11.1)	1 (3.6)	0.552
**Presence of negligence, n (%)**		3 (3.0)	1 (1.8)	0	1 (3.3)	1.000	2 (4.3)	0	2 (7.1)	0.513
**Preoperative shower taken on D0, n (%)**		*n*= 97 62 (63.9)	*n*= 53 39 (73.6)	16 (66.7)	n= 29 23 (79.3)	0.299	n= 44 23 (52.3)	2 (11.1)	n= 26 21 (80.8)	**<0.001**
**Preoperative antibiotic prophylaxis: suitable molecule, n (%)**		*n*= 96	*n*= 53	*n*= 23		0.434	*n*= 43	*n*= 16	*n*= 27	
		95 (99.0)	52 (98.1)	22 (95.7)	30 (100)		43 (100)	16 (100)	27 (100)	
**Operating theatre, n (%)**						0.947	*n*= 44	*n*= 17		0.697
	**1 North**		38 (70.4)	17 (70.8)	21 (70.0)		0	0	0	
	**2 North**		16 (29.6)	7 (29.2)	9 (30.0)		0	0	0	
	**1 South**		0	0	0		11 (25.0)	5 (31.3)	6 (21.4)	
	**2 South**		0	0	0		17 (38.6)	5 (31.3)	12 (42.9)	
	**3 South**		0	0	0		10 (22.7)	3 (18.8)	7 (25.0)	
	**4 South**		0	0	0		6 (13.6)	3 (18.8)	3 (10.7)	
**Position in the daily surgical schedule, n (%)**		*n*= 98				0.234	*n*= 44	*n*= 16		0.780
	**1**	36 (36.7)	26 (48.1)	9 (37.5)	17 (56.7)		10 (22.7)	4 (25.0)	6 (21.4)	
	**2**	39 (39.8)	18 (33.3)	9 (37.5)	9 (30.0)		21 (47.7)	9 (56.3)	12 (42.9)	
	**3**	13 (13.3)	4 (7.4)	3 (12.5)	1 (3.3)		9 (20.5)	3 (18.8)	6 (21.4)	
	**4**	6 (6.1)	4 (7.4)	3 (12.5)	1 (3.3)		2 (4.5)	0	2 (7.1)	
	**5**	4 (4.1)	2 (3.7)	0	2 (6.7)		2 (4.5)	0	2 (7.1)	
**Number of people present in the operating theatre, n (%)**		*n* = 99				0.532	*n*= 45	*n*= 17		0.477
	**4**	1 (1.0)	0	0	0		1 (2.2)	0	1 (3.4)	
	**5**	4 (4.0)	3 (5.6)	3 (12.5)	0		1 (2.2)	1 (5.9)	0	
	**6**	10 (10.1)	6 (11.1)	2 (8.3)	4 (13.3)		4 (8.9)	3 (17.7)	1 (3.6)	
	**7**	22 (22.2)	14 (25.9)	8 (33.3)	6 (20.0)		8 (17.8)	4 (23.5)	4 (14.3)	
	**8**	29 (29.3)	13 (24.1)	6 (25.0)	7 (23.3)		16 (35.6)	4 (23.5)	12 (42.9)	
	**9**	13 (13.1)	6 (11.1)	2 (8.3)	4 (13.3)		7 (15.6)	3 (17.7)	4 (14.3)	
	**10**	11 (11.1)	7 (13.0)	2 (8.3)	5 (16.7)		4 (8.9)	2 (11.8)	2 (7.1)	
	**11**	5 (5.1)	3 (5.5)	1 (4.2)	2 (6.7)		2 (4.4)	0	2 (7.1)	
	**12**	1 (1.0)	1 (1.8)	0	1 (3.3)		0	0	0	
	**13**	1 (1.0)	0	0	0		1 (2.2)	0	1 (3.6)	
	**14**	1 (1.0)	0	0	0		1 (2.2)	0	1 (3.6)	
	**15**	1 (1.0)	1 (1.8)	0	1 (3.3)		0	0	0	
**Presence of a postoperative haematoma, n (%)**		*n*= 86 26 (30.2)	*n*= 47 13 (27.7)	*n*= 20 8 (40.0)	*n*= 27 5 (18.5)	0.104	*n*= 39 13 (33.3)	*n*= 15 4 (26.7)	*n*= 24 9 (37.5)	0.485
**Postoperative anticoagulation, n (%)**		*n*= 93 88 (94.6)	*n*= 50 47 (94.0)	*n*= 20 20 (100)	27 (90.0)	0.265	*n*= 43 41 (95.4)	*n*= 15 15 (100)	26 (92.9)	0.535
**Non-compliance with postoperative recommendations, n (%)**		*n*= 75 3 (4.0)	*n*= 42 1 (2.4)	*n*= 12 1 (8.3)	0	0.286	*n*= 33 2 (6.1)	*n*= 5 1 (20.0)	1 (3.6)	0.284
**Time between surgery and the SSI (days), median (Q1-Q3)**		29.5 (20 – 104)	21.5 (14-48)	19 (11-38)	29 (14-68)	0.136	32 (18-68)	32.5 (17 – 77)	32 (22-63.5)	0.773
**Monomicrobial *S. aureus* SSI, n (%)**	**Superficial**	4 (4.0)	1 (1.9)	0	1 (3.3)	0.286	3 (6.5)	2 (11.1)	1 (3.6)	1.000
	**Deep**	26 (26.0)	13 (24.1)	10 (41.7)	3 (10.0)		13 (28.3)	7 (38.9)	6 (21.4)	
**CCFM SSI, n (%)**	**Superficial**	4 (4.0)	1 (1.9)	0	1 (3.3)	0.381	3 (6.5)	2 (11.1)	1 (3.6)	1.000
	**Deep**	39 (39.0)	20 (37.0)	13 (54.2)	7 (23.3)		19 (41.3)	10 (55.5)	9 (32.1)	
**All SSI, n (%)**	**Superficial**	10 (10.0)	3 (5.6)	1 (4.2)	2 (6.7)	0.585	7 (15.2)	3 (16.7)	4 (14.3)	1.000
	**Deep**	90 (90.0)	51 (94.4)	23 (95.8)	28 (93.3)		39 (84.8)	15 (83.3)	24 (85.7)	

Legend: *ASA* American Society of Anesthesiologists, *BMI* Body mass index, *CCI* Charlson comorbidity index, *CCFM* Cutaneous commensal flora microorganisms, *HBP* High blood pressure, *IV* Intravenous, OS Osteosynthesis, *SSI* Surgical site infection, *UEF* Upper end of the femur

#### Screening data

In the intervention group, among the 2,305 surgical procedures analysed, the preoperative screening result was available for 1,382 (60.0%) surgical procedures. Among these screenings, 24.4% (337/1,382) were positive for MSSA and 0.9% (12/1,382) for MRSA. Note that 21 screenings were carried out in the historical control group and in the North control group, including 10 positive screenings. Regarding the efficacy of decolonisation on the eradication of the carriage of *S. aureus*, 29.8% (107/359) had screening performed perioperatively, of which 91.6% (98/107) were negative.

### Main objective

**Fig. 3:**
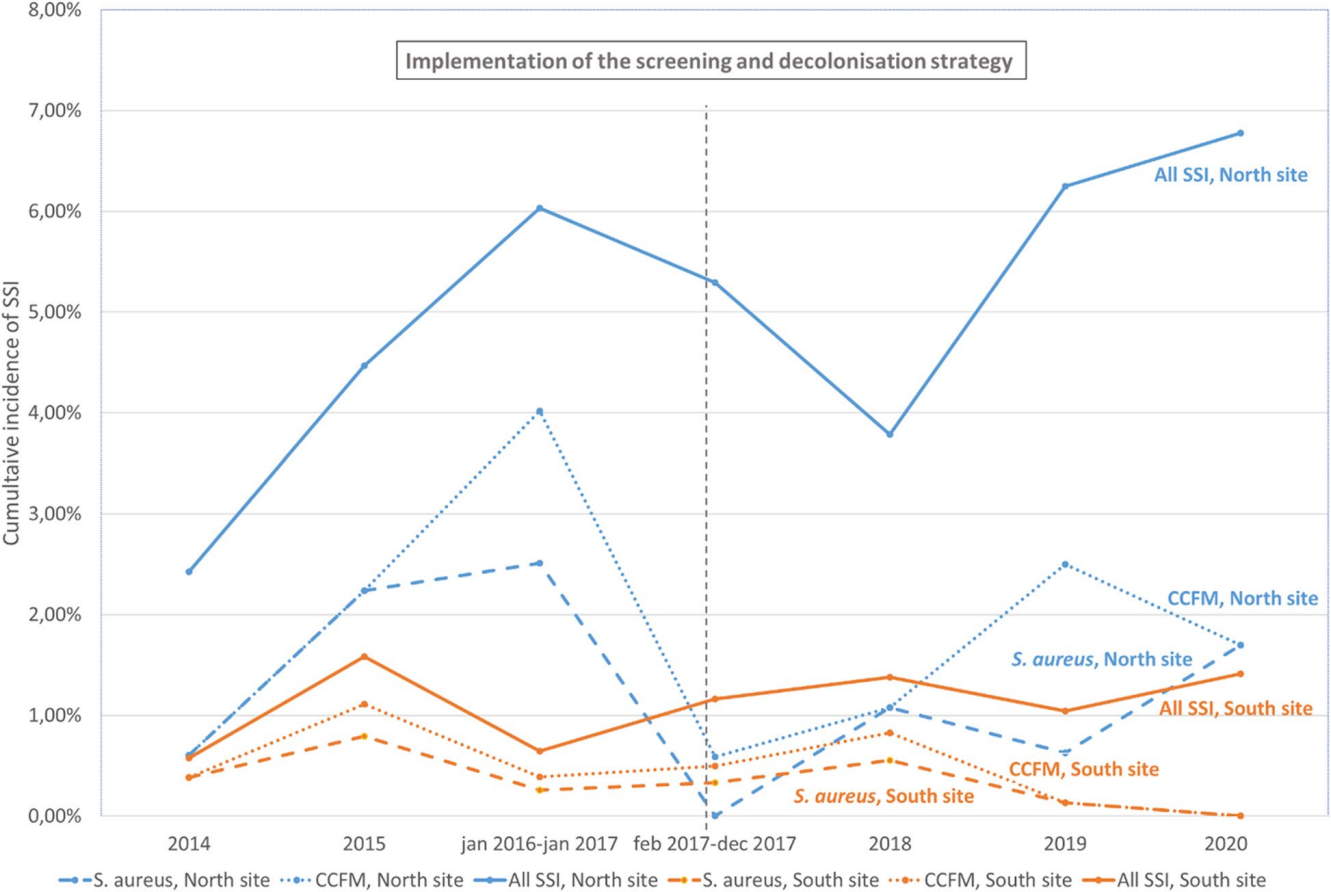
Cumulative incidence rates of SSI after scheduled surgical procedures at the South and North sites. Legend: SSI = surgical site infection, CCFM = cutaneous commensal flora microorganisms

There was no significant difference in the rates of *S. aureus* SSI between the intervention group (7/2,305; 0.3% 95%CI 0.1-0.6) and the historical control group (9/1,926; 0.5% 95%CI 0.2-0.9) (Table 1). Within the South site, the slope of the regression line of *S. aureus* SSI rates per year was negative but not significantly different from 0 (slope=-0.001; *p*=0.175) (Fig. 3). There was a significant difference in the rates of *S. aureus* SSI between the intervention group and the North control group (7/2,305 or 0.3% 95%CI 0.1-0.6 vs. 14/1,117 or 1.3% 95%CI 0.7-2.1 respectively; *p*<0.001). For the South site, the rate of *S. aureus* SSI seems higher in the population of the historical control group that was not screened (9/1,892 or 0.48% 95%CI 0.3-0.9) than in the screened populations of the intervention group whether the result is positive or negative (4/1,382 or 0.29% 95%CI 0.1-0.7) and not screened of the intervention group (3/908 or 0.33% 95%CI 0.1-1.0) (Table 3).

**Table 3:** Description of the preoperative screenings for *S. aureus* carried out within the south site

**South Site**	**Period before**	**Period after**				
	**Not screened** ***n*= 1892**	**Screened positive and negative** ***n*= 1382**		**Screened negative** ***n*= 1033**	**Screened positive** ***n*= 349**	**Not screened *n*= 908**
	***P value***					
**Monomicrobial**	9 (0.48%)	4 (0.29%)	0.408	3 (0.29%)	1 (0.29%)	3 (0.33%)
***S. aureus*** **SSI** ***n*= 16**						
**CCFM SSI** ***n***= **22**	12 (0.64%)	6 (0.44%)	0.451	5 (0.49%)	1 (0.29%)	4 (0.44%)
**All SSI regardless of microorganism** ***n*= 46**	18 (0.95%)	18 (1.30%)	0.351	14 (1.36%)	4 (1.15%)	10 (1.10%)

Legend: *CCFM* Cutaneous commensal flora microorganisms, *SSI* Surgical site infection

### Secondary objectives

There was no significant difference in the rates of CCFM SSI (10/2,305 or 0.4% 95%CI 0.2-0.8 vs. 12/1,926 or 0.6% 95%CI 0.4-1.1) and all SSI regardless of microorganisms (28/2,305 or 1.2% 95%CI 0.8-1.8 vs. 18/1,926 or 0.9% 95%CI 0.6-1.5) between the intervention group and the historical control group (Table 1). Within the South site, the slopes of the regression lines of SSI rates per year were not significantly different from 0: it was negative for CCFM SSI (slope=-0.001; *p*=0.157) and positive for all SSI (slope=0.001; *p*=0.525) (Fig. 3).

There was a significant reduction between the intervention group and the North control group for CCFM SSI (10/2,305 or 0.4% 95%CI 0.2-0.8 vs. 21/1,117 or 1.9% 95%CI 1.2-2.9; *p*<0.001) and for all SSI regardless of microorganisms (28/2,305 or 1.2% 95%CI 0.8-1.8 vs. 54/1,117 or 4.8% 95%CI 3.7-6.3; *p*<0.001).

Within the North site, the slopes of the regression lines of the SSI rates per year were not significantly different from 0: the slope of *S. aureus* SSI was negative (slope=-0.001; *p*=0.592), the slope of CCFM SSI was positive (slope=0.001; *p*=0.843) and the slope of all SSI regardless of microorganisms was positive (slope=0.005; *p*=0.188) (Fig. 3).

### Risk factors for SSI

Patients having an *S. aureus* SSI were compared with patients that did not have an SSI after surgery (Table 4). The risk factors found in the bivariate analysis were male gender (*p*=0.012), a high BMI (*p*=0.010), a high CCI (*p*=0.003), a long surgical time (*p*=0.001) and osteosynthesis procedures (*p*=0.009). Being operated on the South site was a protective factor for *S. aureus* SSI (*p*<0.001). In multivariate analysis, the variables included were BMI, the CCI, the length of the surgery and preoperative screening. The variables for gender, type of surgery and North/south site were excluded from the final model because they did not reach the significance threshold. With this model including 2,609 surgical procedures and 29 SSI, a high BMI (adjusted OR (ORa)_per unit of BMI_, 1.05; 95%CI, 1.0-1.1), a high CCI (ORa_per point of the index_, 1.34; 95%CI, 1.0–1.8) and a long surgical time (ORa_per minute_, 1.01; 95%CI, 1.00–1.02) were significant risk factors for the occurrence of *S. aureus* SSI; preoperative screening (ORa, 0.24; 95%CI, 0.08–0.73) was a significant protective factor.

**Table 4:** Risk factors of *S. aureus* surgical site infections (SSI)

**Variables**		**Monomicrobial** ***S. aureus* SSI** ***n*= 30**	**Surgical procedures without SSI** ***n*= 5248**	**Odds Ratio** **(CI 95%)**	***P* value**	**Adjusted Odds Ratio (95% CI)**	***P* value**
**Age in years, median (Q1-Q3)**		64.5 (54 – 70)	68 (60 – 76)	0.98 (0.96 – 1.01)	0.119		
**Gender, n (%)**	**Male**	20 (0.9)	2303 (99.1)	ref.	**0.012**		
	**Female**	10 (0.3)	2945 (99.7)	**0.39 (0.183 – 0.84)**			
**BMI in kg/m2, median (Q1-Q3)**		*n*= 30	*n*= 2614	**1.05**	**0.010**	**1.05 (1.0 – 1.1)**	**0.049**
		30.7 (28.1-41.8)	27.5 (23.2 – 32.5)	**(1.01 – 1.10)**			
**CCI, median (Q1-Q3)**		1 (0 – 2)	0 (0 – 1)	**1.36 (1.11 – 1.67)**	**0.003**		**0.034**
**ASA score, n (%)**			*n*= 4539		0.273		
	**1**	4 (0.45)	878 (99.5)	ref.			
	**2**	15 (0.56)	2658 (99.4)	1.24 (0.41 - 3.74)			
	**3 and 4**	11 (1.1)	1003 (98.9)	2.41 (0.78 – 7.82)			
**Altemeier classification, n (%)**	**1**	29 (0.5)	5209 (99.5)	ref.	0.205		
	**2**	1 (2.5)	39 (97.5)	4.61 (0.61 – 34.73)			
**Hospitalisation duration before the surgery in hours, median (Q1-Q3)**		18 (17 – 21)	17 (16 – 19)	0.99 (0.99 – 1.02)	0.132		
**Duration of surgery in minutes, median**			*n*= 5275				
**(Q1-Q3)**		81 (67 – 118)	68 (52 – 88)	**1.01 (1.01 – 1.02)**	**0.001**	**1.01 (1.00 – 1.02)**	**0.006**
**Type of surgery, n (%)**	**Hip (primary arthroplasty and revision)**	8 (0.4)	2245 (99.6)	ref.	**0.009**		
	**OS (of the UEF and others)**	7 (1.7)	396 (98.3)	**4.9 (1.79 – 13.76)**			
	**Knee (primary arthroplasty and revision)**	15 (0.6)	2607 (99.4)	1.61 (0.68 – 3.82)			
**Site, n (%)**	**North**	14 (1.3)	1063 (98.7)	ref.	**<**		
	**South**	16 (0.4)	4185 (99.6)	**0.29 (0.14 – 0.60)**	**0.001**		
**Period of surgery,**	**Period before**	19 (0.8)	2427 (99.2)	2.01 (0.95 - 4.32)	0.061		
**n (%)**	**Period after**	11 (0.4)	2821 (99.6)	ref.			
**Presence of preoperative screening, n (%)**		*n*= 29	*n*= 5159		0.112	**0.24 (0.08 – 0.73)**	**0.012**
**Yes** **No**		4 (0.29) 25 (0.66)	1387 (99.71) 3772 (99.34)	0.44 (0.15 – 1.25) ref.			
**Result of preoperative screening, n (%)**		*n*= 4	*n*= 1387		1.000		
**Positive**		1 (0.28)	354 (99.7)	0.97 (0.10 - 9.40)			
**Negative**		3 (0.29)	1033 (99.7)	ref.			
**Result of perioperative screening**^a^, n (%)		*n*= 1	*n*= 106		1.000		
**Positive**		1 (10.0)	9 (90.0)				
**Negative**		0	97 (100)				

Legend: Note that 21 preoperative screenings were carried out outside the intervention group and were included in this table

*ASA* American Society of Anesthesiologists, *BMI* Body mass index, *CCI* Charlson comorbidity index, *95% CI* Confidence interval at 95%, *OS* Osteosynthesis, *Q1-Q3* Quartile1- Quartile3, *SSI* Surgical site infection, *UEF* Upper end of the femur

^**a**^Perioperative screening = control screening among patients with a positive preoperative screening

The multivariate analysis of the risk factors for *S. aureus* SSI for the South site gives the same trends in results as the global analysis with ORa at 0.29 (95%CI, 0.08-1.01) and a p-value at 0.051 for the presence of preoperative screening variable but the other variables are not always significant due to a lack of power (data not shown).

## Discussion

In our population of patients who underwent scheduled orthopaedic surgery, there was no significant decrease in the rates of monomicrobial *S. aureus* SSI (0.3% or 7/2,305 vs. 0.5% or 9/1,926), in the period where the strategy for *S. aureus* screening and decolonisation was implemented compared to the period before implementation. The multivariate analysis at the patient level revealed that the presence of preoperative screening for *S. aureus* was a protective factor.

The global rate of *S. aureus* SSI in our study was 0.6% (30/5,348) and 0.3% (7/2,305) in the intervention group only, which is comparable to French rates according to RAISIN data in 2018 (0.5%) [2]. These results are also comparable to the results of other studies, although there are variations. In three studies, the rates of SSI were respectively 0.4%, 2.7% and 0.45% in the groups without decolonisation and 0.2%, 1.6% and 0.19% in the groups with decolonisation [16–18].

The results of the studies on the *S. aureus* screening and decolonisation in orthopaedic surgery were heterogeneous. Most of the studies were lacking in power. Indeed, Rohrer *et al*. [16] calculated that 15,000 patients were required to obtain sufficient power to demonstrate the interest of this measure in a population where 35% of the patients were *S. aureus* carriers, and with a global rate of SSI of 0.4% in pre-intervention. Four randomised controlled studies evaluated a similar strategy of decolonisation for *S. aureus* in orthopaedic surgery. Three of them did not show any significant result in the rate of *S. aureus* SSI [16, 17, 19]; one study [20] in 2010, which included 917 medicine and general surgery patients, showed a significant protective effect of the decolonisation on all-cause *S. aureus* infections and on *S. aureus* SSI but it was not significant for the subgroup analysis of orthopaedic surgery patients. A prospective pre-post study [21] published in 2015 showed for 31,701 orthopaedic surgery patients in 16 hospitals, a significant effect of the targeted decolonisation in the intervention group (Rate Ratio, 0.48; 95%CI, 0.29-0.80). The results of the retrospective studies are also heterogeneous, but several have shown a significant impact of a screening and decolonisation strategy [22–28]. A meta-analysis conducted in 2020 (7 retrospective studies and 2 prospective studies) on primary knee and hip arthroplasties showed a significant reduction in the risk of *S. aureus* SSI in patients in the screening and decolonisation group compared to the control group (OR, 0.43, 95%CI 0.31-0.59) [29]. Another meta-analysis conducted in 2020 on knee and hip arthroplasties (9 retrospective studies and 1 randomised study) showed a relative risk of SSI of 1.71 (95%CI, 1.34-2.08) and *S. aureus* SSI of 2.79 (95%CI, 1.78-3.81) in the absence of decolonisation [30]. To our knowledge, there is no meta-analysis including only the randomised controlled studies performed in orthopaedic surgery. Eventually, the strong association between the screening strategy and the risk of SSI in our study might have been overestimated for several reasons such as the existence of confounding factors that were not included in the analyses or a potential indication bias (patients with a lower risk of SSI more likely to be screened).

The independent significant risk factors of *S. aureus* SSI found in our study in multivariate analysis were the BMI, the CCI and the length of the surgery. Preoperative screening and targeted decolonisation was a significant independent protective factor. We were not able to collect certain known risk factors of *S. aureus* SSI such as smoking and the absence of a preoperative shower for all patients. The patient characteristics in the North control group were different as compared to those of the South group, in particular in terms of Altemeier classification, ASA scores, lengths of surgery, and proportion of surgical procedures for osteosyntheses, but North/South site variable was not included in the multivariate analysis because it did not modify the significance of the result. These results are coherent with those of literature; an analysis of 3,618 *S. aureus* SSI after knee and hip prosthesis procedures [31] found the following risk factors: male gender, length of surgery >120 minutes, ASA score ≥2 and hip prosthesis replacement. Diabetes, smoking, nasal carriage of *S. aureus*, cancer, the NNIS score and the BMI were also risk factors found in some orthopaedic surgery studies [9, 32, 33].

In our study, screening was carried out only at the nasal level. Even though it is the most frequent colonisation site, other colonisation sites have been described (throat, axillae, perineum, etc.) [34]. Some *S. aureus* carriers might not have been correctly identified and decolonised, therefore underestimating the impact of the strategy. Moreover, the decolonisation regimen by Mupirocin and Chlorhexidine has been shown to be associated with a lower eradication rate for *S. aureus* for patients colonised regardless of the colonisation site (71.9%) compared to patients who were positive for the nasal screening alone (92%) [34]. A preoperative screening of different sites could improve the impact of the strategy.

In our institution, we implemented a targeted decolonisation strategy. Most cost-efficacy studies show a reduction in the costs associated with decolonisation. The reduction in the SSI rate and the economic gain seem more substantial with the universal decolonisation strategy compared to targeted decolonisation [30]. A pre-post study published in 2016 [35], including 4,186 surgical procedures, compared a targeted decolonisation strategy with a universal decolonisation strategy. The rate of *S. aureus* SSI had dropped significantly after implementing universal decolonisation (0.09% vs 0.5%; p = 0.01). The economic gain was around $700K over the 25 months of the universal decolonisation period.

We did not study the bacterial resistance to Mupirocin and Chlorhexidine in our study. A recent meta-analysis showed a rate of resistance of *S. aureus* to Mupirocin of 6.6% in Europe [36]. The use of Mupirocin seems associated with an increase in resistance, although certain studies are contradictory [37], and the high levels of resistance to Mupirocin are correlated with decolonisation failures [38]. Studies on the prevalence of reduced sensitivity to Chlorhexidine are very few, and the results are heterogeneous with prevalence varying between 0.6% and 70% [39]. The use of Chlorhexidine could be associated with an increase in the strains with reduced sensitivity [40], but some studies did not observe this association [41]. Alternative treatments for the decolonisation of *S. aureus* have been studied, in particular povidone-iodine as an intranasal ointment, but the studies still seem very few in number to recommend its use in common practice [42].

Our study has several limits. Firstly, this study was retrospective. Most of the data were automatically extracted from electronic patient records. However, the data regarding screening and decolonisation was not automated for patients who were not screened in our institution. The laboratory performing the screening was asked to systematically send the results to the hospital, but we cannot exclude any failures in the traceability of this information. Secondly, we cannot exclude a lack of power. Indeed, despite a substantial inclusion period (6 and a half years), only 100 SSI of which 30 monomicrobial *S. aureus* SSI occurred among the 5,348 surgical procedures that were monitored. Thirdly, the observance of the decolonisation was not assessed for all patients in our study. It cannot be excluded that a portion of the *S. aureus* carriers did not undergo, or incompletely, the decolonisation treatment, resulting in a decrease in the impact of the strategy. However, among the patients colonised with *S. aureus* who had a perioperative control screening, 92% (97/106) were negative. These results are in line with several studies [17, 34, 43] and are in favour of a relatively good observance of the treatment. Moreover, this reflects real-life conditions more than a randomised controlled study.

Despite the low number of SSI in our substantial cohort of patients, the screening and targeted decolonisation of S*. aureus* carriers was a protective factor of *S. aureus* SSI after scheduled orthopaedic surgery. These results encourage us to continue the strategy of screening and decolonisation in our centre in order to increase our study population. Although the cost-efficacy studies are currently in favour of a universal decolonisation strategy, the emergence of resistance to Mupirocin and to a lesser degree to Chlorhexidine, have to be taken into account in the decision to use these molecules on a wide scale.

## Data Availability

No datasets were generated or analysed during the current study.

## Abbreviations

ASA: American Society of Anesthesiologists
BMI: body mass index
CDC: centers for disease control and prevention
CHUGA: centre hospitalier universitaire Grenoble Alpes
CCI: Charlson comorbidity index
CoNS: Coagulase negative *Staphylococci*
CI: Confidence interval
CCFM: Cutaneous commensal flora microorganisms
HBP: High blood pressure
IV: Intravenous
MRSA: Methicillin-resistant *Staphylococcus aureus*
MSSA: Methicillin-sensitive *Staphylococcus aureus*
NNIS: National nosocomial infections surveillance
OR: Odds ratio
OS: Osteosynthesis
RAISIN: Réseau d'alerte, d'investigation et de surveillance des infections nosocomiales
SSI: Surgical site infection
UEF: Upper end of the femur
WHO: World health organization
